# Serum and Dietary Vitamin D in Individuals with Class II and III Obesity: Prevalence and Association with Metabolic Syndrome

**DOI:** 10.3390/nu13072138

**Published:** 2021-06-22

**Authors:** Erika Aparecida Silveira, Camila Kellen de Souza Cardoso, Letícia de Almeida Nogueira e Moura, Ana Paula dos Santos Rodrigues, Cesar de Oliveira

**Affiliations:** 1 Department of Epidemiology & Public Health, Institute of Epidemiology & Health Care, University College London, London WC1E 6BT, UK; c.oliveira@ucl.ac.uk; 2 Postgraduate Program in Health Sciences, Faculty of Medicine, Federal University of Goiás, Goiânia 74690-900, Brazil; camilacardoso_nut@hotmail.com (C.K.d.S.C.); leticiaanm@gmail.com (L.d.A.N.e.M.); anapsr@gmail.com (A.P.d.S.R.); 3 Nutrition Course, Catholic Pontifices University of Goiás, Goiânia 74605-010, Brazil; 4 Municipal Hospital of Aparecida of Goiânia, Aparecida de Goiânia 74936-600, Brazil; 5 Goias State Health Department, Goiânia 74093-250, Brazil

**Keywords:** obesity, metabolic syndrome, 25-Hydroxyvitamin D, diabetes mellitus, HDL cholesterol, hypertension, diet, aging

## Abstract

The association between vitamin D deficiency and metabolic syndrome (MS) in severe obesity is unclear and controversial. We analyzed serum and dietary vitamin D and their association with MS in 150 adults with class II and III obesity (BMI ≥ 35 kg/m^2^) from the DieTBra Trial (NCT02463435). MS parameters were high fasting blood glucose, low HDL cholesterol, high triglycerides, elevated waist circumference, and hypertension. Vitamin D deficiency was considered as a level < 20 ng/mL. We performed multivariate Poisson regression adjusted for sociodemographic and lifestyle variables. The prevalence of serum vitamin D deficiency was 13.3% (mean 29.9 ± 9.4 ng/mL) and dietary vitamin D median was 51.3 IU/day. There were no significant associations between vitamin D, serum, and diet and sociodemographic variables, lifestyle, and class of obesity. Serum vitamin D deficiency was associated with age ≥ 50 years (*p* = 0.034). After a fully adjusted multivariate Poisson regression, MS and its parameters were not associated with serum or dietary vitamin D, except for lower HDL, which was associated with serum vitamin D deficiency (PR = 0.71, 95% CI 0.52–0.97; *p* = 0.029). Severe obese individuals had a low prevalence of vitamin D deficiency, which was not associated with MS.

## 1. Introduction

The prevalence of obesity has been increasing, and it represents a global public health concern [1]. Obesity classes II and III have been rising more substantially than class I in several countries globally [2,3,4]. In the USA, severe obesity, which includes both class II and III, affects 9.3% of adults [5], and it has been projected to affect 24.2% in 2030 [3]. In Brazil, this prevalence is 1.7% of its adult population [2]. The presence of obesity is also associated with other non-communicable chronic diseases, as well as reduced life expectancy [6,7]. In the context of the SARS-VOC-2 pandemic, obese and severely obese individuals have a higher risk of severe disease and mortality [8]. In addition, obesity can increase the risk of hypovitaminosis D through different physiological mechanisms and also due to the lower sun exposure of these individuals in outdoor physical activities [9]. Vitamin D deficiency influences energy metabolism, favoring the development of obesity [10,11] and it is also implicated in reduced immune response [8,11].

Adiposity is directly related to increases in pro-inflammatory cytokines, which in turn collaborate with insulin resistance and favor the occurrence of dyslipidaemia [12] and increases in the risk of cardiovascular diseases, some types of cancer, and type 2 diabetes [13,14]. Adipose and metabolic tissue disorders in obese individuals contribute to metabolic syndrome (MS) [15] as well as to hypovitaminosis D [16]. Serum vitamin D levels are negatively correlated with MS’s parameters, such as abdominal obesity, insulin resistance, fasting blood glucose, arterial hypertension, and triglycerides [17,18,19,20]. In fact, the association between MS and vitamin D has been studied in several populations [21,22,23]. In children, a potentially harmful relationship was observed between hypovitaminosis D and risk of obesity [24]. Research carried out with older adults found that low levels of vitamin D were a risk factor for MS [25].

However, in individuals with severe obesity, there are still few studies analyzing vitamin D deficiency and MS, with conflicting results [26,27,28,29]. In adults with class III obesity (BMI ≥ 40 kg/m^2^), there was a negative correlation between BMI and vitamin D [26]. A study carried out with 73 Caucasian adults showed an association between hypovitaminosis D and MS in individuals with severe obesity [28]. On the other hand, another study with adults with severe obesity (mean BMI of 45 kg/m^2^) found no significant association between MS parameters and serum vitamin D levels before undergoing bariatric surgery [27]. Therefore, the association between vitamin D deficiency and MS in this group is not fully understood. The present study aims to estimate the profile of serum and dietary vitamin D in class II/III obese individuals and to evaluate the association of serum and dietary vitamin D with MS and its parameters. The hypothesis of the study is that individuals with class II/III obesity have a high prevalence of serum vitamin D deficiency that is associated with MS and some of its parameters.

## 2. Methods

### 2.1. Study Design and Participants

This study analyzed baseline data from the clinical trial “Effect of nutritional intervention and olive oil in severe obesity—DieTBra Trial”, registered on the ClinicalTrials.gov platform (NCT02463435), carried out at the Nutrition Clinic for Severe Obesity at the Clinics Hospital, Federal University of Goiás (HC/UFG), Goiânia, Goiás, Brazil. As the present study included information of one point of time and before any intervention, it is characterized as cross-sectional. Individuals who were diagnosed with class II or III obesity in the SUS (Unified Health System) were advised to go to the Nutrition Clinic for Severe Obesity at the Clinics Hospital (HC/UFG) to receive more specific treatment. For the individuals who attended the ambulatory service, we have applied the eligibility criteria described below. More details about the study design and recruitment can be found in previous publications [30,31,32,33,34,35,36]. All participants signed an informed consent form to participate in the study, in accordance with the Declaration of Helsinki. The study was approved by the Research Ethics Committee (CEP/HC/UFG), protocol number 747.792.

Adults aged between 18 and 65 years, from both sexes, with a body mass index (BMI) ≥ 35 kg/m^2^, living in the metropolitan region of Goiânia, capital of Goiás state, Midwest Brazil, were included. The following exclusion criteria were adopted: pregnancy, breastfeeding, people with physical disability (hearing, speaking, or walking), and individuals who had already had bariatric surgery, were receiving nutritional treatment, or had weight loss > 8% in the last three months. Regarding medication, daily use and previous diagnoses were also excluded: corticosteroids and anti-inflammatory drugs, individuals with cancer, HIV/AIDS, obstructive pulmonary disease, liver or kidney failure, and cardiac insufficiency.

### 2.2. Sociodemographic Data, Lifestyle, and Anthropometry

Sociodemographic and lifestyle data were obtained using a standardized and previously tested questionnaire. The sociodemographic variables evaluated were sex, age, skin color, level of education, and social class according to the Brazilian Economic Classification Criteria of the Brazilian Association of Research Companies [37]. This method to classify social class in Brazil is based on the physical structure of the household, possession of goods and equipment, as well as the level of education of the head of the family. For each condition, there is a score, and each class is defined by the sum of that score [37]. Skin color is a proxy for race/ethnicity largely used in epidemiological and clinical studies in Brazil. The participants have auto-declared their skin color.

The lifestyle variables assessed were smoking status (smoker, ex-smoker, and never smoked) [38] and alcohol consumption assessed in episodes of excessive drinking, determined by consumption on a single occasion of ≥5 doses for men and ≥4 doses for women [39].

Weight and height were used to calculate the body mass index (BMI = weight (kg)/height (m)^2^. Body weight was measured on a digital platform scale with a capacity of 200 kg and accuracy of 100 g (Welmy ^®^) For height, a stadiometer coupled to the digital scale was used, with 0.1 cm precision and performed according to standardized techniques [40].

The measurement of abdominal circumference was obtained through multi-frequency electrical bioimpedance using the Inbody S10^®^ device (Biospace Co. Ltd.a, Seoul, Korea), which has six different frequencies—1, 5, 50, 250, 500, and 1000 khz. The examination was performed with the patient in an orthostatic position while fasting. Instructions were given to participants not to perform physical activity or drink alcoholic beverages on the day before the test. Due to the absence of specific cut-off points for this population, the classification of a high waist circumference was based on the 75th percentile, i.e., ≥124.6 cm in women and ≥129.9 cm in men.

### 2.3. Clinical Variables and Vitamin D Intake

Biochemical tests were performed after a 12-h fast. Blood glucose, HDL cholesterol, and triglycerides were measured by colorimetric enzymatic method, and vitamin D was analyzed using the electrochemiluminescence method. Glycaemia (fasting plasma glucose) was classified as high when ≥100 mg/dL or when the patient was using a hypoglycemic medication [41]. HDL cholesterol was classified as low when <40 mg/dL for men or <50 mg/dL for women or when using a lipid-lowering medication [42]. Triglycerides were classified as high when ≥150 mg/dL or when using medication to treat hypertriglyceridemia [42].

Blood pressure was measured with the individual at rest and seated, with the arm at heart level and with an appropriate cuff-size for obese individuals. A semi-automatic device was used (Omron HEM 742INT, Omron Healthcare Inc., Kyoto, Japan). The procedure was repeated twice, at a 3 min interval, and the average of the two measurements was calculated [43]. Blood pressure was classified as high when ≥130/85 mmHg or when using antihypertensive medication [43].

For the MS classification, the simultaneous presence of at least three of the following conditions was considered: elevated waist circumference, high triglycerides, high blood pressure, high fasting plasma glucose, and low HDL-cholesterol [42]. Vitamin D intake was assessed by the average of three 24-h dietary recalls method, two in person and one via telephone contact. In the 24-h dietary methods, the individuals described to the dietitian all foods and beverages intake on the day before the interview. After that, the dietitian calculated the macro and vitamin D intake based on the table of food constitution [44,45]. Vitamin D intake was categorized considering the 50th percentile and the cut-off point in this study was of 70.4 IU/day. We have used the Avanutri online nutritional software to calculate the 24 h recalled food intake.

### 2.4. Statistical Analysis

The database was built using the EPI DATA^®^ program (version 3.5.1, EpiData Association, Odense, Denmark), with double entry for subsequent analysis of consistency and quality. Statistical analyses were performed on STATA 12 SE (Stata Corp, College Station, TX, USA). At first, the Kolmogorov–Smirnov test was applied to check the normality of the data. In the descriptive analysis, absolute and relative frequency and prevalence, in addition to mean and median, were calculated. In the bivariate analysis, chi-square or Fisher’s exact tests were used to compare proportions and the Mann–Whitney or Kruskal–Wallis tests were used to compare medians.

Multiple Poisson regression was performed using three adjustment models. Model 1 with sociodemographic variables (gender, age, education, and socioeconomic class), model 2 (model 1 + smoking status and alcoholic consumption), and model 3 (model 1 + model 2 + BMI). All variables with a value of *p* < 0.20 in the bivariate analysis were included in the multivariate analysis. *p* < 0.05 was considered statistically significant.

## 3. Results

Among the 150 individuals with severe obesity who participated in this research, the mean serum vitamin D was 29.9 ± 9.4 and a median of 28.75 ng/mL. The prevalence of insufficient serum vitamin D was 40% (median: 77.1 IU/day) and deficient 13.3% (median: 51.3 IU/day), with no significant difference between categories (*p* = 0.189). For vitamin D, based on food consumption data, the median intake was 70.4 IU/day.

Regarding the sociodemographic and lifestyle variables, 85.3% were women, 38% were aged 30 to 39 years old, 61% belonged to the socioeconomic class C, 67.3% never smoked, and 54.4% reported not having episodes of excessive drinking. BMI between 40 and 49.9 kg/m^2^ was observed in 56.7% of individuals (Table 1).

In the analysis of the association between serum vitamin D with sociodemographic, lifestyle, and anthropometric variables, only age was significant (*p* = 0.051), whereas for dietary vitamin D, no statistically significant associations were observed with any of the variables included in the study (Table 1). These analyses were also performed using the median values, and the only significant association was with age and serum vitamin D (*p* = 0.047) (Table S1, Supplementary Material). Additional results are presented as Supplementary Material.

There was no association between serum and dietary vitamin D with MS’s parameters. The prevalence of MS in class II/III obese participants was 69.3% (Table 2). Analyses of serum and dietary vitamin D according to MS’s parameters were also performed using the median, but no statistically significant associations were found (Table S2, Supplementary Material).

In the fully adjusted multivariate regression models, serum and dietary vitamin D were not statistically associated with metabolic syndrome and its parameters (Table 3 and Table 4). The only exception was the significant association with low HDL that protected against serum vitamin D deficiency around 30% (PR = 0.71, 95% CI 0.53–0.97). These analyses were also performed with serum vitamin D using a < 30 ng/mL cut-off point, and no significant associations were found (Table S3, Supplementary Material).

## 4. Discussion

This is one of the few studies carried out exclusively with class II/III obese individuals (BMI > 35 kg/m^2^) evaluating the association of serum and dietary vitamin D with metabolic syndrome. In adult individuals with class II/III obesity, the prevalence of serum vitamin D deficiency in the present study was 13.3% lower than expected. Despite the various statistical approaches we have used, there was no significant association between serum or diet with MS in the unadjusted and fully adjusted models for sociodemographic, lifestyle, and anthropometric covariates. Only the low HDL cholesterol was associated as a protective factor for serum vitamin D deficiency. Therefore, our main findings bring important contributions to this research area, which has not been much studied, with conflicting results [27,28].

In the present study, the prevalence of vitamin D deficiency (13.3%) was lower than an observational study carried out in the city of Rio de Janeiro, in which the prevalence in adults with class III obesity was 35.3% [26]. Our prevalence was also lower than the Spanish study with adults with a BMI ≥ 40 kg/m^2^ (50.7%) [28] and a study with patients in the pre-operative period of bariatric surgery (72%) [27]. The lower prevalence observed in the present study can be justified by the higher incidence of sunlight in the Brazilian Midwest region where the study was conducted. In addition, our analysis was also performed with a good sample size when compared to the other studies mentioned above. In obese people, the high prevalence of vitamin D deficiency can be explained by lifestyle and health variables such as less exposure to sunlight, low consumption of food sources of this vitamin, and impaired skin synthesis [46]. In addition, the fat-soluble characteristic of vitamin D, which has a greater affinity for adipose tissue, makes this vitamin sequestered and retained in adipocytes, reducing its bioavailability and circulating concentration [47,48]. A recent study has hypothesized that the absorption of fat-soluble vitamins, including D, decrease in individuals with metabolic syndrome. However, the authors found that the presence of metabolic syndrome did not affect the vitamin D status [49].

In our study, there was no association between MS or its parameters with dietary vitamin D. Data from the 2003–2006 National Health and Nutrition Examination Surveys (NHANES), with 3543 participants aged ≥ 20 years, showed that those in the highest quartile of intake were 28% less likely to have MS (OR = 0.72; 95% CI 0.58–0.90) when compared to the lowest intake quartile [50]. In the aforementioned study, the median intake was 48.7 IU/day in the second and 100.7 IU/day in the third quartile [50], while in the present study, it was 70.4 IU/day. In the present study, vitamin D intake values were further lower of the estimated average requirement for healthy individuals of 400 IU/day [51].

There is a discussion about the difficulty of reaching the recommendation of adequate intake (RDA) of 600 IU/day, proposed by the daily recommended intake (DRIs) of vitamin D only through food intake [51,52]. This high recommendation leads to the high prevalence of inadequate vitamin D intake observed in epidemiological or clinical studies. In tropical countries like Brazil, with high sunlight incidence all year, this recommendation of dietary intake does not seem appropriate, especially in the Midwest region of Brazil where the present study was carried out and with a high incidence of sun all year round. Perhaps it would not even be necessary to follow these intake recommendations in countries like Brazil, since sun exposure can be the main source of vitamin D in tropical countries. DRI’s recommendations are more appropriate to Nordic countries; thus, it is necessary to develop more appropriate guidelines for tropical and sub-tropical countries. These recommendations also depend on individual characteristics such as health, age, weight, diet, and cultural habits, making regional or national guidelines more applicable both at public health level and individually in clinical practice [51,53].

Surprisingly, we did not observe an association between vitamin D and SM, even after using different cut-off points and statistical approaches. This result differs from the study carried out with individuals with severe obesity, in which vitamin D deficiency was a predictor of MS [28]. However, our results were similar to another three studies with severely obese individuals, which also observed that vitamin D deficiency was not associated with MS [27,29], including one conducted in Brazilian adults [26]. It is well known that abdominal obesity and obesity are the main risk factors of MS [54,55]. The association between vitamin D deficiency and obesity has been discussed in the scientific literature [9,47,48], but with controversial results. Our study corroborates studies that did not observe an association between vitamin D and MS or its parameters [29,56,57].

The individual MS parameters in our sample were also not associated with vitamin D deficiency, which is different from the study of European Caucasians, which found that vitamin D deficiency was associated with a higher concentration of triglycerides (*p* = 0.015). Our study, however, is in agreement with research in individuals with severe obesity in the preoperative period of bariatric surgery, who also found no association between components of MS and vitamin D deficiency [27].

In our study, we observed that low HDL cholesterol protected around 30% of vitamin D deficiency, which differs from studies with adults with morbid obesity that did not observe an association between vitamin D deficiency and HDL [26,29]. However, some studies found a similar result [28,58,59] including a longitudinal analysis of the Atherosclerosis Risk in Communities (ARIC) data with 5.2 years of follow-up, which showed that individuals with vitamin D deficiency had a greater reduction in HDL levels compared to those with adequate concentrations [58]. A recent systematic review and meta-analysis of studies with adults, selected regardless of their nutritional status, found a significant effect of vitamin D on cholesterol, TG, and LDL cholesterol, but not on HDL [60]. This association has only occurred with serum vitamin D and not dietary vitamin D, probably due to the quality of information from serum vitamin Dm i.e., biochemical analysis is more accurate then estimation of dietary intake.

The mechanism of vitamin D influencing the lipid profile, in particular HDL, is still unclear. Some hypotheses from previous studies indicated that hypovitaminosis D may be associated with impaired pancreatic B cell function and insulin resistance, which can modify the metabolism of lipoproteins, such as the reduction of HDL [59,61,62]. Vitamin D-dependent metabolic pathways interact more with HDL than with other lipid particles, as there is a stronger positive association between 25(OH)D and larger HDL particles, indicating a possible vitamin D action in the regulation of reverse cholesterol transport [58,63].

We observed that serum vitamin D decreased with increasing age, even in our study with only adult individuals. No previous studies were found that analyzed this association between vitamin D and age in adults with class II/III obesity. We found only one study that observed an association between age and vitamin D, but the sample included both adults and adolescents with class II/III obesity; however, the higher prevalence of vitamin D deficiency was in adolescents [26]. An association between age and vitamin D deficiency was observed in older adults, but obesity was not associated with this condition [60]. With increasing ageing, there is a decrease in vitamin D receptors, renal production of 1,25(OH)2D, and cutaneous synthesis of 7-dehydrocholesterol, resulting in a 50% reduction in the pre-vitamin D3 as well as substrates involved in this production [64,65].

As a possible limitation of the present study, we can mention the assessment of dietary vitamin D with a food consumption 24 h recall method. This instrument depends on the participant’s memory and report, which may lead to some memory bias. However, this type of bias is expected when using these instruments, and to minimize this effect, an average of three 24 h periods (72 h) has been employed [44,45,66] and the interviewers were well-trained. The sample size can also be considered a potential limitation when compared with large population studies. On the other hand, by being a sample exclusively of individuals with a BMI > 35 kg/m^2^, it is an expressive sample when compared with other studies with individuals with this degree of obesity [27,28].

The results of the present study are relevant and provide additional information on serum and dietary vitamin D in individuals that are still not much studied, i.e., class II/III obesity. This vitamin acts in several physiological processes, but only few studies have been conducted specifically with this population group. Despite vitamin D being associated with several risk factors for various health conditions, we did not find an association between vitamin D deficiency and MS. These results can be quite useful both in the research area and clinical practice for the different treatment scenarios of individuals with severe obesity.

## 5. Conclusions

Severely obese individuals had a low prevalence of vitamin D deficiency, i.e., less than 13.5%. Serum and dietary vitamin D were not associated with MS or its diagnostic parameters, except for low HDL cholesterol levels, which were a protective factor for serum vitamin D deficiency. Thus, in individuals with class II/III obesity, vitamin D was not a risk factor for MS, as well as the other parameters or comorbidities used for the diagnosis of MS.

## Figures and Tables

**Table 1:** Serum and dietary vitamin D and sociodemographic, lifestyle, and anthropometric variables in individuals with class II/III obesity.

Variables	*n* (%)	Vitamin D Serum		Vitamin D Dietary	
		<20 ng/mL	*p*	<70.4 IU/Day (p50)	*p*-Value
**Sex**					
Men	22 (14.7)	2 (10.0)	0.739 *	8 (10.7)	0.166 **
Women	128 (85.3)	18 (90.0)		67 (89.3)	
Age groups (years)					
18–29	19 (12.7)	1 (5.0)	0.051 *	11 (14.7)	0.708 **
30–39	57 (38.0)	4 (20.0)		28 (37.3)	
40–49	53 (35.3)	9 (45.0)		24 (32.0)	
≥ 50	21 (14.0)	6 (30.0)		12 (16.0)	
Skin colour					
White	46 (30.7)	5 (25.0)	0.367 *	20 (26.7)	0.491 **
Brown	83 (55.3)	14 (70.0)		45 (60.0)	
Black	21 (14.0)	1 (5.0)		10 (13.3)	
Schooling years					
≤4	15 (10.0)	3 (15.0)	0.521 *	6 (8.0)	0.619 **
5–11	110 (73.3)	13 (65.0)		55 (73.3)	
≥ 12	25 (16.7)	4 (20.0)		25 (18.7)	
Social Class					
A–B	34 (22.7)	3 (15.0)	0.543 *	19 (25.3)	0.400 **
C	92 (61.3)	15 (75.0)		42 (56.0)	
D–E	24 (16.0)	2 (10.0)		14 (18.7)	
Smoking					
Never	101 (67.3)	13 (65.0)	0.811 **	53 (70.7)	0.384 **
Ex-smoker/smoker	49 (32.7)	7 (35.0)		22 (29.3)	
Binge drinking					
Yes	43 (54.4)	3 (30.0)	0.093 *	27 (61.4)	0.165 **
No	36 (45.6)	7 (701.0)		17 (38.6)	
BMI (kg/m^2^)					
35–39.9	25 (16.7)	4 (20.0)	0.212 *	17 (22.7)	0.141 **
40–49.9	85 (56.7)	8 (40.0)		39 (52.0)	
≥ 50	40 (26.6)	8 (40.0)		19 (25.3)	

* Fisher. ** chi-square. p50 (median). Binge drinking was calculated based on 79 drinkers.

**Table 2:** Vitamin D (serum and dietary) according to metabolic syndrome parameters in individuals with class II/III obesity.

Variables	*n* (%)	Vitamin D Serum		Vitamin D Dietary	
		<20 ng/mL *n* = 20 (13.3%)	*p* Value *	<70.4 IU/Day (p50) *n* = 75 (50%)	*p* Value *
Glycaemia					
≥100 mg/dL or medication	69 (46.0)	9 (45.0)	0.923	34 (45.3)	0.870
<100 mg/dL	81 (54.0)	11 (55.0)		41 (54.7)	
HDL-cholesterol					
<40 mg/dL or <50 mg/L or medication	82 (54.7)	11 (55.0)	0.974	42 (56.0)	0.743
≥40 mg/dL or ≥50 mg/L	68 (45.3)	9 (45.0)		33 (44.0)	
Triacylglycerol					
≥150 mg/dL or medication	72 (48.0)	9 (45.0)	0.773	36 (48.0)	1.000
<150 mg/dL	78 (52.0)	11 (50.0)		39 (52.0)	
Abdominal circumference					
≥129.9 or ≥124.6 cm	37 (25.5)	7 (35.0)	0.295	19 (27.1)	0.664
<129.9 cm or 124.6 cm	108 (74.5)	13 (65.0)		51 (72.9)	
Elevated blood pressure					
≥130/85 mmHg or medication	94 (62.7)	15 (75.0)	0.321	45 (60.0)	0.500
<130/85 mmHg	56 (37.3)	5 (25.0)		30 (40.0)	
Metabolic Syndrome					
Yes	104 (69.3)	15 (75.0)	0.615	52 (69.3)	1.000
No	46 (30.7)	5 (25.0)		23 (30.7)	

* chi-square or fisher. p50 (median).

**Table 3:** Multiple regression analyses of the association between serum vitamin D with metabolic syndrome and its parameters in class II/III obesity.

Variables	Model 1 Vitamin D Serum (<20 ng/mL)			Model 2 Vitamin D Serum (<20 ng/mL)			Model 3 Vitamin D Serum (<20 ng/mL)		
	PR	CI 95%	*p* *	PR	CI 95%	*p* *	PR	CI 95%	*p* *
Glycaemia									
<100 mg/dL	1			1			1		
≥100 mg/dL or medicament	1.055	0.734–1.515	0.773	0.991	0.556–1.781	0.976	0.945	0.521–1.712	0.852
HDL-cholesterol									
≥40 mg/dL or ≥50 mg/L	1			1					
<40 mg/dL or <50 mg/L or medicament	0.713	0.526–0.966	0.029	0.775	0.522–1.149	0.205	0.732	0.488–1.097	0.131
Triacylglycerol									
<150 mg/dL	1			1			1		
≥150 mg/dL or medicament	0.962	0.699–1.324	0.814	1.125	0.693–1.827	0.632	0.991	0.589–1.668	0.973
Abdominal circumference									
<129.9 cm or 124.6 cm	1			1	1		-	-	-
≥129.9 or ≥124.6m	1.050	0.939–1.174	0.388	1.086	0.926–1.274	0.308	-	-	-
Blood pressure									
<130/85 mmHg	1			1			1		
≥130/85 mmHg or medicament	0.983	0.768–1.258	0.891	1.109	0.765–1.607	0.586	1.202	0.799–1.807	0.376
Metabolic syndrome									
No	1				1			1	
yes	0.954	0.7649–1.189	0.674	1.091	0.784–1.517	0.606	1.025154	0.727–1.444	0.887

* Poisson regression. PR (prevalence ratio). CI (confidence interval). Model 1: sex, age, schooling years, social class. Model 2: model 1 + smoking e binge drinking. Model 3: model 1 + model 2 + BMI.

**Table 4:** Multiple regression analyses of the association between dietary vitamin D and metabolic syndrome and its parameters in class II/III obesity.

Variables	Model 1 Vitamin D Dietary p50 (<70.4 IU/Day)			Model 2 Vitamin D Dietary p50 (<70.4 IU/Day)			Model 3 Vitamin D Dietary p50 (<70.4 IU/Day)		
	PR	CI 95%	*p* *	PR	CI 95%	*p* *	PR	CI 95%	*p* *
Glycaemia									
<100 mg/dL	1			1			1		
≥100 mg/dL or medicament	0.976	0.693–1.375	0.891	0.906	0.504–1.630	0.743	0.868	0.474–1.588	0.645
HDL–cholesterol lower									
≥40 mg/dL or ≥50 mg/L	1			1			1		
<40 mg/dL or <50 mg/L or medicament	0.972	0.742–1.274	0.840	0.912	0.644–1.291	0.603	0.930	0.654–1.325	0.690
Triacylglycerol									
<150 mg/dL	1			1			1		
≥150 mg/dL or medicament	1.032	0.735–1.451	0.854	1.219	0.736–2.019	0.440	1.2240	0.729–2.057	0.444
Abdominal circumference									
<129.9 cm or 124.6 cm		1			1		–	–	–
≥129.9 or ≥124.6 m	0.969	0.863–1.088	0.596	1.020	0.879–1.184	0.792	–	–	–
Blood pressure									
<130/85 mmHg		1			1			1	
≥130/85 mmHg or medicament	1.043	0.821–1.324	0.729	0.830	0.566–1.218	0.342	0.825	0.558–1.222	0.338
Metabolic syndrome									
No		1			1			1	
yes	0.968	0.784–1.194	0.760	0.837	0.589–1.1882	0.319	0.797	0.564–1.125	0.196

* Poisson regression. PR (prevalence ratio). CI (confidence interval). Model 1: sex, age, schooling years, social class. Model 2: model 1 + smoking e binge drinking. Model 3: model 1 + model 2 + BMI.

## Data Availability

Not applicable.

## Supplementary Materials

The following are available online at https://www.mdpi.com/article/10.3390/nu13072138/s1, Table S1: Serum and dietary vitamin D levels by sociodemographic, lifestyle and anthropometric characteristics in individuals with class II/III obesity, Table S2: Vitamin D, serum and dietary, according to metabolic syndrome parameters in individuals with class II/III obesity. Table S3: Multiple linear regression of serum and dietary vitamin D adjusted by sociodemographic data, anthropometry and metabolic syndrome parameters in severe obese individuals.
