# Supplementary material for: Serum and Dietary Vitamin D in Individuals with Class II and III Obesity: Prevalence and Association with Metabolic Syndrome
Source: Nutrients. 2021 Jun 22;13(7):2138. doi: 10.3390/nu13072138 (PMC8308482; doi:10.3390/nu13072138)
Supplement: Supplementary file 1 [file nutrients-13-02138-s001.zip › nutrients-1234027-supplementary.pdf]

Supplementary Tables

Table S1. Serum and dietary vitamin D levels by sociodemographic, lifestyle and anthropometric characteristics in individuals with class II/III obesity.

**Variables**	**N (%)**	**Vitamin D serum (ng/mL) Median**	**p***	**Vitamin D dietary (IU/day) Median**	**p***
Sex					
Men	22 (14.7)	28.6	0.996	95.1	0.119
Women	128 (85.3)	28.8		67.9	
Age groups (years)					
18-29	19 (12.7)	32.3	**0.047**	61.9	0.711
30-39	57 (38.0)	30.6		73.2	
40-49	53 (35.3)	25.5		73.2	
≥50	21 (14.0)	26.7		51.2	
Skin colour					
White	46 (30.7)	32.3	0.656	86.9	0.864
Brown	83 (55.3)	30.6		66.5	
Black	21 (14.0)	25.5		71.1	
Schooling years					
≤4	15 (10.0)	27.3	0.498	73.2	0.586
5-11	110 (73.3)	28.9		70.2	
≥12	25 (16.7)	27.8		56.4	
Socioeconomic Class					
A-B	34 (22.7)	30.1	0.223	67.1	0.173
C	92 (61.3)	27.3		78.8	
D-E	24 (16.0)	32.1		61	
Smoking status					
Never	101 (67.3)	30.3	0.271	68.1	0.949
Ex-smoker/ smoker	49 (32.7)	27.8		82.7	
Binge drinking #					
Yes	43 (54.4)	29	0.448	58.9	0.335
No	36 (45.6)	27.1		75.1	
BMI (kg/m^2)					
35-39.9	25 (16.7)	27.7	0.197	47.2	0.179
40-49.9	85 (56.7)	30.4		80.9	
≥50	40 (26.6)	25.3		73.2	

**Teste Mann-Whitney ou Kruskal-Wallis. # The "n" in the binge drinking variable is lower than other variables because it just considered those who drink. p50 (median).

Table S2. Vitamin D, serum and dietary, according to metabolic syndrome parameters in individuals with class II/III obesity.

**Variables**	**N (%)**	**Vitamin D serum (ng/mL) Median**	**p***	**Vitamin D dietary (IU/day) Median**	**p****
Glycaemia					
≥100 mg/dL or medication	69 (46.0)	28.8	0.865	71.1	0.837
<100 mg/dL	81 (54.0)	28.7		69.7	
HDL-cholesterol					
<40 mg/dL or <50 mg/L or medication	82 (54.7)	26.6	0.179	69.4	0.872
≥40 mg/dL or ≥50 mg/L	68 (45.3)	30.5		71.2	
Triacylglycerol					
≥ 150 mg/dL or medication	72 (48.0)	28.3	0.943	70.7	0.386
<150 mg/dL	78 (52.0)	29.6		70.4	
Abdominal circumference					
≥129.9 or ≥124.6 cm	37 (25.5)	30.5	0.492	69.7	0.639
<129.9 cm or124.6 cm	108 (74.5)	28.9		74.9	
Elevated blood pressure					
≥130/85mmHg or medication	94 (62.7)	28.3	0.927	73.2	0.656
<130//85 mmHg	56 (37.3)	29.7		66.6	
Metabolic Syndrome					
Yes	104 (69.3)	28.0	0.642	70.4	0.491
No	46 (30.7)	30.0		69.5	

* Mann Whitney Test.

Table S3. Multiple linear regression between serum and dietary vitamin D adjusted by sociodemographic data, anthropometry and metabolic syndrome parameters in severe obese individuals.

Variables	Vitamin D serum		Vitamin D dietary	
	β (95%IC)	p	β (95%IC)	p
Sex	-	-		
Women	-	-	1.00	
Men	-	-	12.957 (-17.0008;42.923)	0.394
Age groups (years)				
18-29	1.00			
30-39	-0.827 (-5.644; 3.991)	0.735	-	-
40-49	-4.799 (-9.655; 0.075)	0.054	-	-
≥50	-6.282 (-12.072; -0.493)	**0.034**	-	-
Socioeconomic Class				
A-B	-	-	1.00	
C	-	-	11.709 (-14.617; 38.037)	0.381
D-E	-	-	-4.8113 (-39.739; 30.117)	0.786
BMI (kg/m^2)				
35-39.9	1.00		1.00	
40-49.9	2.514 (-1.649; 6.678)	0.235	20.602 (-7.666;48.871)	0.152
≥50	-1.203 (-5.909; 3.503)	0.614	11.685 (-20.834;44.205)	0.479
HDL cholesterol				
≥40 mg/dL or ≥50 mg/L	1.00		-	-
<40 mg/dL or <50 mg/L or medication	-1.674 (-4.685; 1.336)	0.273	-	-
Abdominal circumference				
≥129.9 or ≥124.6 cm	-	-	1.00	
<129.9 cm or124.6 cm		-	-75.520 (-200.915;49.873)	0.236
Elevated blood pressure				
<130//85 mmHg	-	-	-	-
≥130/85mmhg or medication	-	-	-	-
Metabolic Syndrome				
No	-	-	-	-
Yes	-	-	-	-

Serum vitamin D adjusted by age, BMI and HDL. Dietary vitamin D adjusted by sex, socioeconomic class, BMI and abdominal circumference.
